# FAK overexpression is correlated with tumour invasiveness and lymph node metastasis in oesophageal squamous cell carcinoma

**DOI:** 10.1038/sj.bjc.6601050

**Published:** 2003-07-01

**Authors:** T Miyazaki, H Kato, M Nakajima, M Sohda, Y Fukai, N Masuda, R Manda, M Fukuchi, K Tsukada, H Kuwano

**Affiliations:** 1 Department of Surgery I, Gunma University Faculty of Medicine, 3-39-22, Showa-machi, Maebashi, Gunma 371-8511, Japan

**Keywords:** oesophageal cancer, focal adhesion kinase, prognosis, differentiation, immunohistochemistry

## Abstract

Focal adhesion kinase (p125^FAK^; ‘FAK’) is a tyrosine kinase that is localised to cellular focal adhesions and is associated with a number of other proteins, such as integrin adhesion receptors. We performed an immunohistochemical analysis of FAK protein expression to determine the relationship between FAK overexpression and clinicopathological factors in oesophageal squamous cell carcinoma (ESCC). We examined tissue specimens that had been removed from 91 patients with thoracic oesophageal cancer who had undergone surgery between 1983 and 2001. Immunohistochemical staining was performed by the standard streptavidin–biotin method. Seven human ESCC cell lines–TE-1, TE-2, TE-8, TE-13, TE-15, TT, and TTn–and one immortalized human keratinocyte cell line–HaCaT–were used in Western blot analysis. Immunostaining of FAK was seen in the cytoplasm of cancer cells, particularly in cells located in the invasive fronts of cancer nests. FAK overexpression was detected in 54 of the 91 patients (59.3%). Significant correlations were observed between FAK overexpression and cell differentiation (*P*=0.0057), depth of tumour invasion (*P*=0.0023), presence of regional lymph node metastasis (*P*=0.0097), number of lymph node metastases (*P*=0.0026), and disease stage (*P*=0.012). The survival rates of patients with FAK-overexpressing cancer were significantly lower than those of patients without FAK-overexpression cancer (*P*=0.006). The 5-year survival rate of patients without FAK overexpression was 69%, whereas that of patients with FAK overexpression was 38%. On Western blot analysis, FAK was expressed at a high level in TE-1, TE-8, TE-15, and TT cells, at a moderate level in TE-2 and TTn cells, and at a low level in TE13 and HaCaT cells. FAK phosphorylation at tyrosine 397 was demonstrated in proportion to the intensity of FAK in all cell lines except TE15 and HaCaT. In conclusion, FAK overexpression of ESCC was related to cell differentiation, tumour invasiveness, and lymph node metastasis. Consequently, patients with ESCC who had FAK overexpression had a poor prognosis.

Cancer invasion and metastasis are complex processes that include changes in cell adhesion, allowing transformed cells to invade and migrate through the extracellular matrix (Liotta *et al*, 1991). These adhesions are mediated in part through the integrin family of cell surface receptors. Integrins are partly localised on the ventral surfaces of clusters called focal adhesions (Burridge *et al*, 1988). Focal adhesion kinase (p125^FAK^, hereafter referred to as FAK), is a tyrosine kinase that is localised to cellular focal adhesions and is associated with a number of other proteins, such as the integrin adhesion receptors (Richardson and Parsons, 1995). The first indication that FAK might be involved in tumorigenesis came from the observation that it was one of several highly tyrosine-phosphorylated proteins in src-transformed fibroblasts (Kanner *et al*, 1990; Schaller *et al*, 1992). In normal cells, FAK is the major tyrosine-phosphorylated protein present in cells upon activation of the integrin receptors (Guan *et al*, 1991). FAK is involved in integrin-signalling pathways (Kornberg *et al*, 1992; Lipfert *et al*, 1992; Schlaepfer *et al*, 1994), cellular motility (Cary *et al*, 1996; Guan, 1997), and apoptosis (Frisch *et al*, 1996; Hungerford *et al*, 1996; Xu *et al*, 1996). Cells derived from pp125^FAK^−/− mouse embryos exhibit reduced migration as a result of impaired adhesion turnover (Ilic *et al*, 1995, 1996). Overexpression of FAK has been reported in a number of invasive human cancer cells (Weiner *et al*, 1993; Akasaka *et al*, 1995; Owens *et al*, 1995; Tremblay *et al*, 1996; McCormack *et al*, 1997; Cance *et al*, 2000). In some of these reports, there is a suspected relationship between FAK expression and metastatic ability.

There have been many studies of FAK expression in cancer cell lines and cancer tissues. However, few investigations have used immunohistochemical analysis, because almost all anti-FAK antibodies are ineffective for staining formaldehyde-fixed paraffin-embedded tissue sections (Cance *et al*, 2000). There have been no reports of FAK expression in oesophageal squamous cell carcinoma (ESCC). We investigated FAK protein expression in ESCC by immunohistochemical analysis using FAK-specific monoclonal antibody 4.47 (Upstate Biotechnology Inc., Lake Placid, NY, USA) (Cance *et al*, 2000). Our aim was to determine the relationship between FAK overexpression and clinicopathological factors in ESCC. Further, we used Western blot analysis to elucidate FAK overexpression and signal transduction in ESCC cell lines.

## MATERIALS AND METHODS

### Patients and tissue samples

The tissue specimens used had been removed from 91 patients with thoracic ESCC who had undergone surgery at the Gunma University Hospital between 1983 and 2001. Written informed consent to participate in the study was obtained from each patient before surgery, according to the ethical guidelines of our university. All patients underwent potentially curative surgery without preoperative therapy. There were 77 men and 14 women, aged 40−78 years (mean age: 61.0 years). Tumour stages were classified according to the 5th edition of the TNM classification of the International Union against Cancer (UICC). The evaluation of tumour differentiation was based on histological criteria of the guidelines of the Japanese Society for Esophageal Diseases (Japanese Society for Esophageal Diseases, 1999). The mean postoperative follow-up period was 33.9 months (range: 6.2−192.2 months).

Specimens were fixed in 10% formaldehyde solution and embedded in paraffin. We examined sections that contained both a tumour-invasive portion and normal oesophageal epithelium.

### Cell lines

Seven human oesophageal cancer cell lines — TE-1, TE-2, TE-8, TE-13, TE-15, TT, and TTn — and one spontaneously immortalised human keratinocyte cell line — HaCaT — were used. The TE cell lines were kindly provided by Dr T Nishihira (Institute of Development, Aging and Cancer, Tohoku University School of Medicine, Sendai, Japan) (Nishihira *et al*, 1993). TT and TTn cells (JCRB0262 and 0261) were kindly provided by Dr K Takahashi (Takahashi *et al*, 1990). All cancer cell lines were derived from ESCC with varying degrees of differentiation (Nishihira *et al*, 1993). TE-1, TE-15, TT, and TTn were well-differentiated squamous cell carcinoma (SCC) in primary lesions. TE-8 was moderately differentiated SCC and TE-2 and TE-13 were poorly differentiated SCC. The TE cell lines were established from surgical specimens of the primary lesions. The TT cell line was obtained directly from a surgical specimen of a metastatic lesion in the mandible. The TTn cell line was established from a transplanted tumour in a nude mouse, the primary lesion being the same as that from which the TT cell line was established. It has been reported that all these cell lines were transplantable in nude mice (Takahashi *et al*, 1990; Galiana *et al*, 1993; Nishihira *et al*, 1993). TE-2, TE-8, and TE-13 have the wild type of p53 (Barnas *et al*, 1997; Itoshima *et al*, 2000; Akimoto *et al*, 2001). Regarding the p53 status of TE-1 and TTn, a mutation at codon 272 was reported, respectively (Barnas *et al*, 1997; Itoshima *et al*, 2000). In TE-15, a heterozygous G-to-A mutation was detected in the splice-acceptor site of intron 5 of p53 (Barnas *et al*, 1997). TE cell lines were cultured in RPMI-1640 medium (Sigma, St Louis, MO, USA) supplemented with 10% foetal bovine serum and antibiotics (100 U ml^−1^ penicillin and 100 *μ*g ml^−1^ streptomycin). TT and TTn were cultured in 1 : 1 Dulbecco's modified Eagle medium (DMEM) and Ham's F-12 medium (Sigma) containing 10% foetal bovine serum and antibiotics, as described above. HaCaT was cultured in DMEM medium (Sigma) containing 10% foetal bovine serum and antibiotics, as described above. All cell lines were cultured to 60−80% confluence.

### Antibodies

Antibodies were purchased from the following manufacturers: monoclonal antibody (Mab) specific for FAK (clone 4.47), (Upstate Biotechnology Inc., Lake Placid, NY, USA); rabbit polyclonal antibody specific for FAK-phosphorylated at tyrosine 397 (FAK[pY^397^]), (BioSource International Inc., Camarillo CA, USA); Mab specific for Ki-67 (MIB-1) (Immunotech, Marseille, France); Mab specific for *β*-actin, Sigma.

### Immunohistochemistry for FAK protein and Ki-67 protein

Immunohistochemical staining was performed by the standard streptavidin–biotin (SAB) method. Briefly, each 4-*μ*m tissue section was deparaffinised, then rehydrated and incubated with fresh 0.3% H_2_O_2_ in methanol for 30 min at room temperature. After rehydration through a graded ethanol series, the sections were autoclaved in 1 mM EDTA buffer (pH 8.0) at 120°C for 5 min for anti-FAK Mab and were autoclaved in 10 mM citrate buffer (pH 6.0) at 120°C for 5 min for anti-Ki-67 FAK Mab , then cooled to 30°C. After incubation with normal rabbit serum for 30 min, the tissue sections were removed by blotting. The sections were then incubated at 4°C overnight with anti-FAK Mab at a dilution of 1 : 1000 in phosphate-buffered saline (PBS) containing 1% bovine serum albumin, then washed in PBS and incubated with secondary antibody for 30 min at room temperature. Immunohistochemistry was performed using a Histofine SAB-PO(M) kit (Nichirei, Tokyo, Japan). The chromogen was 3,3′-diaminobenzidine tetrahydrochloride, applied as a 0.02% solution containing 0.0055% H_2_O_2_ in 50 mM ammonium acetate–citric acid buffer (pH 6.0). The sections were lightly counterstained with hematoxylin. Negative controls were prepared by substituting normal mouse serum for primary antibody, and no detectable staining was evident.

### Evaluation of immunostaining for FAK and Ki-67 labelling index

When >40% of carcinoma cells in a given specimen were stained more intensely than the normal epithelium in the same section, the sample was classified as FAKoverexpression (FAK(+)). Ki-67 labelling index was calculated as the percentage of nuclear staining of cells at the invasive front of the tumour in three consecutive high-powered fields (× 400); each field corresponded to a total number of cells ranging from 300 to 1000. We counted at least 1000 cells per sample.

### Cell extraction and Western blotting

Lysates from exponentially growing cell lines were prepared in buffer (20 mM Tris-HCl, pH 7.6, 1 mM EDTA, 140 mM NaCl, 1% Nonidet P-40, 1% aprotinin, 1 mM phenylmethylsulphonyl fluoride, 1 mM sodium vanadate). The protein concentration was determined using a BCA Protein Assay Kit (Pierce, Rockford, IL, USA). Protein (30 *μ*g) from each cell line was resuspended in sodium dodecyl sulphate (SDS) sample buffer (100 mM Tris-HCl, pH 8.8, 0.01% bromophenol blue, 36% glycerol, 4% SDS) containing 1 mM dithiothreitol, boiled for 5 min, and subjected to a 5−10% gradient Ready-Gel (Bio-Rad, Tokyo, Japan). Proteins were electrotransferred to a Hybond-enhanced chemiluminescence nitrocellulose membrane (Amersham Pharmacia Biotech, Buckinghamshire, UK). Proteins were immunoblotted by using anti-FAK (clone 4.47; Upstate Biotechnology). The bands were detected using an enhanced chemiluminescence detection system (Amersham Pharmacia Biotech). For reblotting; membranes were stripped according to the manufacturer's protocol. Proteins were reblotted using anti-FAK[pY^397^] and anti-*β*-actin (Sigma). Anti-*β*-actin (Sigma) antibody served as the control.

### Statistical analysis

Statistical analysis was performed using the unpaired two-group *t*-test for age, number of lymph node metastases and Ki-index. The *χ*^2^ test was used for gender, differentiation, location, and TNM clinical classification. Survival curves were calculated by the Kaplan–Meier method, and analysis was carried out by the log-rank test.

## RESULTS

### Relationship between FAK expression and clinicopathological features

**Figure 1:**
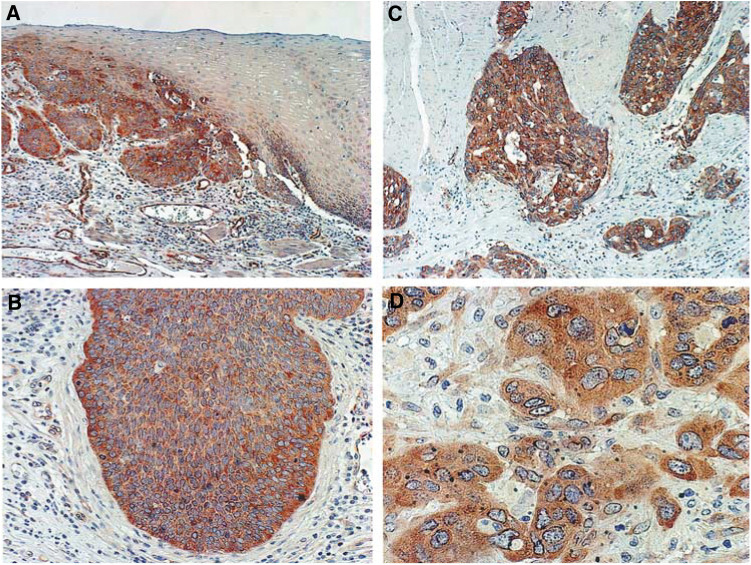
Representative photomicrographs of tissue sections immunostained for FAK. (**A**) FAK was detected in the cytoplasm of the basal cells, parabasal cells, and leukocytes in normal oesophageal epithelium (right). Primary oesophageal cancer with FAK protein overexpression (× 100) (left). This case was regarded as FAK-overexpression (+). (**B**) FAK protein overexpression was detected in invasive cancer fronts, particularly in cells located in the peripheral layers of cancer cell nests (× 200). (**C**) Scattering small clusters of cancer cells have expressed FAK protein abundantly (× 100). (**D**) High-power view of the immunohistochemistry. FAK was detected in the cytoplasm of cancer cells (× 400).

FAK expression in ESCC was investigated by immunohistochemical analysis of formalin-fixed, paraffin-embedded specimens using a FAK-specific Mab. In normal oesophageal tissue, immunostaining of FAK was detected in the cytoplasm of the basal cells, parabasal cells, vascular endothelial cells, and leukocytes (Figure 1A

). Immunostaining of FAK was seen in the cytoplasm of all cancer cells, particularly in cells located in the invasive fronts of the cancer nests (Figure 1B–D). As heterogeneous expression of FAK was noted in tumours, samples were classified as FAK overexpression (+) when >40% of carcinoma cells were stained more intensely than the normal epithelial basement membrane. FAK overexpression was detected in 54 of the 91 patients (59.3%). The relationship between the clinicopathological characteristics of patients with ESCC and FAK overexpression is summarized in Table 1

**Table 1:** Correlation between clinicopathological characteristics and FAK expression

		**FAK(−)**	**FAK(+)**	
**Parameters**	**Total**	***n*=37**	***n*=54**	***P*-value**
Age (mean±s.d. years)	61.0±8.2	59.8±8.2	61.5±7.9	0.052
Gender	77	34	43	
Male	14	3	11	0.11
Female				
Differentiation				
Well	23	13	10	
Moderate	45	21	24	
Poorly	23	3	20	0.0057
Location				
Upper	12	6	6	
Mid-thoracic	56	21	35	
Lower	23	10	13	0.69
*TNM clinical classification*				
T				
T1	35	20	15	
T2	13	8	5	
T3	37	9	28	
T4	6	0	6	0.0023
N				
N0	37	21	16	
N1	54	16	38	0.0097
M	75	32	43	
M0	16	5	11	0.40
M1				
Stage				
I	24	15	9	
II	28	13	5	
III	23	4	19	
IV	16	5	11	0.012
No. of lymph node metastasis (mean±s.d.)		0.78±1.2	3.3±4.8	0.0026
Ki index (mean±s.d.)	—	37.8±16.7	42.4±16.0	0.18
Total	91	37	54	

FAK(+)=FAK overexpression (+); FAK(−)=FAK overxpression (−); s.d.=standard deviation.

. A significant correlation was observed between FAK overexpression and cell differentiation (*P*=0.0057), depth of tumour invasion (*P*=0.0023), presence of regional lymph node metastasis (*P*=0.0097), number of lymph node metastases (*P*=0.0026), and disease stage (*P*=0.012). However, there was no significant association with age, sex, tumour location, or presence of distant metastasis.

It was reported that integrin signalling through FAK leads to the regulation of cell proliferation and survival (Schlaepfer *et al*, 1994; Guan, 1997). Ki-67 is a useful marker for evaluating the proliferation potential of normal and tumour cells. Therefore, we studied the correlation between the expression of FAK and the Ki-67-labelling index. The mean index in FAK-overexpression (+) patients was 42.4±16.0, and higher than that in FAK-overexpression (−) patients (37.8±16.7). However, the difference was not significant (*P*=0.18).

**Figure 2:**
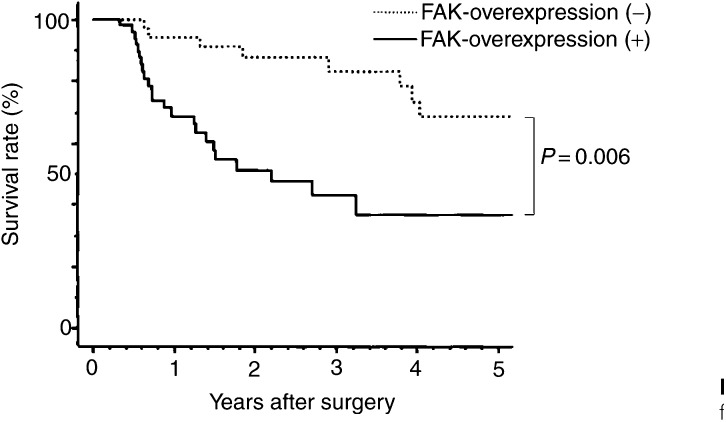
Relationship between overall postoperative survival and FAK expression. FAK-overexpression (−) patients had a significantly more favourable prognosis than those with FAK overexpression (5-year survival rates: FAK overexpression (+), 38%; FAK overexpression (−), 69%; *P*=0.006).

The survival rates of patients with FAK-overexpression (+) cancer were significantly lower than those of patients with FAK-overexpression (−) cancer (*P*=0.006; Figure 2

). The mean 5-year survival rate of patients without FAK overexpression was 69%, whereas that of patients with FAK overexpression was 38%. Multivariate analysis showed that FAK overexpression was not a prognostic factor by itself, in contrast to depth of tumour invasion, lymph node metastasis, or disease stage (data not shown). We reanalysed the prognosis of patients with the same pathological background. The survival rates of patients with FAK-overexpressing cancer were lower than those of patients without FAK-overexpressing cancer for those at stages T1N0, T1N1, T2N0, T2N1, and T3N1, but the difference was not significant. There was no difference in survival for T3N0 patients.

### FAK expression at the protein level and FAK phosphorylation at tyrosine 397 in the cell lines

**Figure 3:**
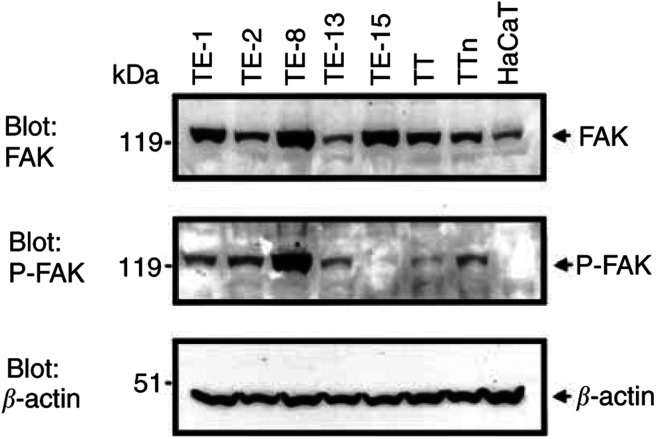
Western blotting of cell extracts from seven ESCC lines. The figure shows the expression of FAK: 125 kDa; FAK phosphorylated at tyrosine 397 (P-FAK): 125 kDa and *β*-actin: 42 kDa (control).

The expression of FAK at the protein level was investigated in seven cell lines derived from ESCC and one immortalised human keratinocyte cell line. Western blotting revealed different levels of expression of FAK (Figure 3

; upper panel). FAK was expressed at a high level in TE-1, TE-8, TE-15, and TT cells, at a moderate level in TE-2 and TTn cells, and at a low level in TE-13 and HaCaT cells.

Tyrosine 397 was identified as a major site of FAK autophosphorylation (Schaller *et al*, 1994). To investigate the catalytic activity of FAK in each cell line, immunoblotting with anti-FAK[pY^397^] was performed (Figure 3). FAK phosphorylation at tyrosine 397 was demonstrated in proportion to the intensity of FAK in all cell lines except TE-15 and HaCaT. FAK phosphorylation at tyrosine 397 existed at a very low level in TE-15 cells, and there was no endogenous expression of FAK[pY^397^] in HaCaT cells.

## DISCUSSION

Our immunohistochemical results suggest that the expression of FAK protein is correlated with cell differentiation, depth of tumour invasion, occurrence of regional lymph node metastasis, and the number of lymph node metastases. FAK overexpression was detected in all cancer cells. In particular, strong expression was observed in invasive tumour fronts. This result indicated that invading cancer cells expressed FAK abundantly. The proliferation activity of tumour with FAK overexpression is higher than that without FAK overexpression, which is not significant statistically. Furthermore, the prognosis of patients who overexpressed FAK was significantly less favourable than that of FAK-overexpression (−) patients. A number of reports have indicated that FAK may be up-regulated in human tumour cells of diverse origin (Weiner *et al*, 1993; Akasaka *et al*, 1995; Owens *et al*, 1995; Tremblay *et al*, 1996; McCormack *et al*, 1997; Cance *et al*, 2000). In some of these reports, there has been a suspected relationship between FAK expression and metastatic ability (Owens *et al*, 1995; Tremblay *et al*, 1996). These observations support the results of our study. Slack *et al* demonstrated that an increase in FAK expression, coupled with tyrosine phosphorylation of FAK on tyrosine 861, may contribute to the increased cell motility of highly tumorigenic prostate cancer cells (Slack *et al*, 2001). Overexpression of FAK may alter the motility of ESCC cells, and as a result of this high motility, FAK protein might be overexpressed in those patients with more invasive and metastatic ESCC. Further work is clearly required to investigate the relationship between FAK overexpression and tumour malignancy in ESCC.

We investigated the levels of expression of FAK in seven ESCC cell lines and one immortalised human keratinocyte cell line. All ESCC cell lines expressed various levels of FAK and, except for TE-13, the levels were higher than those in the immortalised human keratinocyte cell line. Although immunohistochemical analysis showed a significant correlation between FAK overexpression and poor tumour differentiation, there was no relationship between FAK expression and cell line differentiation. This discrepancy may have been due to differences between the characteristics of the cell lines and the pathological diagnoses of the primary lesions. There was no relationship between FAK overexpression and p53 status in the ESCC cell lines. Galiana *et al* (1993) have reported that the TE-1, TE-2, and TE-8 cell lines are more highly tumorigenic than TE-13 in nude mice. Our results showed that the FAK level was high in TE-1 and TE-8, intermediate in TE-2, and low in TE-13. From our results, we suspected that overexpression of FAK might be correlated with high tumorigenicity in human ESCC cell lines. In all but one of the ESCC cell lines, the level of FAK phosphorylation at tyrosine 397 was similar to the amount of FAK protein. It is interesting that in TE-15, we detected a low level of phosphorylation at tyrosine 397 of FAK in spite of the abundance of FAK protein. We cannot explain this result, although it might have been caused by the characteristics of TE-15. As the signal intensity of FAK in all cell lines was similar to the amount of FAK protein, we consider that the evaluation of FAK expression might lead indirectly to an evaluation of FAK signal transduction.

It has been reported that the *fak* gene dosage is increased in a variety of cell lines derived from SCC of the head and neck, lung, breast, and colon cancer (Agochiya *et al*, 1999).^25^ As our study material was ESCC, the observed overexpression of FAK might have been caused by *fak* gene amplification.

In our study, patients with FAK overexpression had a poor prognosis for overall survival. However, multivariate statistical analysis showed that FAK overexpression was not a prognostic factor by itself. Therefore, our result was presumably influenced by the factors of cell differentiation, tumour invasion, and lymph node metastasis. Some reports have demonstrated that the degree of cell differentiation is a useful prognostic factor in ESCC patients (Torres *et al*, 1999; Wang *et al*, 1999). Invasion and metastasis, the main causes of death in most cancer patients, remain the most important but least understood aspects of cancer. In particular, the presence of lymph node metastasis and the number of nodal metastases are associated with a poor prognosis in oesophageal cancer (Kuwano *et al*, 1997; Altorki and Skinner, 2001). As FAK overexpression was related to these factors, patients with FAK overexpression had a poor prognosis.

Detection of FAK expression in formaldehyde-fixed paraffin-embedded tissue sections by immunohistochemical techniques is easy, low cost, and quantifiable, and reveals the localisation of FAK overexpression. Intense FAK expression in preoperative biopsy specimens may be an indicator of advanced disease with a high probability of tumour spread. FAK may be a good therapeutic target, the manipulation of which may prevent ESCC cells from invading other organs and spreading into the lymphatic drainage.

In conclusion, FAK overexpression is related to cell differentiation, tumour invasiveness, and lymph node metastasis. Consequently, patients whose tumours overexpress FAK have a poorer prognosis than those whose tumours do not.

## References

[bib1] Agochiya M, Brunton VG, Owens DW, Parkinson EK, Paraskeva C, Keith WN, Frame MC (1999) Increased dosage and amplification of the focal adhesion kinase gene in human cancer cells. Oncogene 18: 5646–5652 10523844 10.1038/sj.onc.1202957

[bib2] Akasaka T, van Leeuwen RL, Yoshinaga IG, Mihm Jr MC, Byers HR (1995) Focal adhesion kinase (p125FAK) expression correlates with motility of human melanoma cell lines. J Invest Dermatol 105: 104–108 7615962 10.1111/1523-1747.ep12313396

[bib3] Akimoto T, Nonaka T, Ishikawa H, Sakurai H, Saitoh JI, Takahashi T, Mitsuhashi N (2001) Genistein, a tyrosine kinase inhibitor, enhanced radiosensitivity in human esophageal cancer cell lines *in vitro*: possible involvement of inhibition of survival signal transduction pathways. Int J Radiat Oncol Biol Phys 50: 195–201 11316564 10.1016/s0360-3016(00)01560-1

[bib4] Altorki N, Skinner D (2001) Should en bloc esophagectomy be the standard of care for esophageal carcinoma? Ann Surg 234: 581–587 11685019 10.1097/00000658-200111000-00001 PMC1422081

[bib5] Barnas C, Martel-Planche G, Furukawa Y, Hollstein M, Montesano R, Hainaut P (1997) Inactivation of the p53 protein in cell lines derived from human esophageal cancers. Int J Cancer 71: 79–87 9096669 10.1002/(sici)1097-0215(19970328)71:1<79::aid-ijc14>3.0.co;2-4

[bib6] Burridge K, Faith K, Kelley T, Nuckolls G, Turner C (1988) Focal adhesions: transmembrane junctions between the extracellular matrix and the cytoskeleton. Annu Rev Cell Biol 4: 487–525 3058164 10.1146/annurev.cb.04.110188.002415

[bib7] Cance WG, Harris JE, Iacocca MV, Roche E, Yang X, Chang J, Simkins S, Xu L (2000) Immunohistochemical analysis of focal adhesion kinase expression in benign and malignant human breast and colon tissues: correlation with preinvasive and invasive phenotypes. Clin Cancer Res 6: 2417–2423 10873094

[bib8] Cary LA, Chang JF, Guan JL (1996) Stimulation of cell migration by overexpression of focal adhesion kinase and its association with Src and Fyn. J Cell Sci 109: 1787–1794 8832401 10.1242/jcs.109.7.1787

[bib9] Frisch SM, Vuori K, Ruoslahti E, Chan-Hui PY (1996) Control of adhesion-dependent cell survival by focal adhesion kinase. J Cell Biol 134: 793–799 8707856 10.1083/jcb.134.3.793 PMC2120934

[bib10] Galiana C, Fusco A, Martel N, Nishihira T, Hirohashi S, Yamasaki H (1993) Possible role of activated ras genes in human esophageal carcinogenesis. Int J Cancer 54: 978–982 8335405 10.1002/ijc.2910540619

[bib11] Guan JL (1997) Role of focal adhesion kinase in integrin signaling. Int J Biochem Cell Biol 29: 1085–1096 9416004 10.1016/s1357-2725(97)00051-4

[bib12] Guan JL, Trevithick JE, Hynes RO (1991) Fibronectin/integrin interaction induces tyrosine phosphorylation of a 120-kDa protein. Cell Regul 2: 951–964 1725602 10.1091/mbc.2.11.951 PMC361893

[bib13] Hungerford JE, Compton MT, Matter ML, Hoffstorm BG, Otey CA (1996) Inhibition of p125^FAK^ in cultured fibroblasts results in apoptosis. J Cell Biol 135: 1383–1390 8947559 10.1083/jcb.135.5.1383 PMC2121083

[bib14] Ilic D, Furuta Y, Kanazawa S, Takeda N, Sobue K, Nakatsuji N, Nomura S, Fujimoto J, Okada M, Yamamoto T (1995) Reduced cell motility and enhanced focal adhesion contact formation in cells from FAK-deficient mice. Nature 377: 539–544 7566154 10.1038/377539a0

[bib15] Ilic D, Kanazawa S, Furuta Y, Yamamoto T, Aizawa S (1996) Impairment of mobility in endodermal cells by FAK deficiency. Exp Cell Res 222: 298–303 8598217 10.1006/excr.1996.0038

[bib16] Itoshima T, Fujiwara T, Waku T, Shao J, Kataoka M, Yarbrough WG, Liu TJ, Roth JA, Tanaka N, Kodama M (2000) Induction of apoptosis in human esophageal cancer cells by sequential transfer of the wildtype p53 and E2F-1 genes: involvement of p53 accumulation via ARF-mediated MDM2 down-regulation. Clin Cancer Res 6: 2851–2859 10914734

[bib17] Japanese Society for Esophageal Diseases (1999) Guidelines for the Clinical and Pathological Studies on Carcinoma of the Esophagus, 9th edn, pp 43 Tokyo: Kanehara

[bib18] Kanner SB, Reynolds AB, Vines RR, Parsons JT (1990) Monoclonal antibodies to individual tyrosine phosphorylated protein substrates of oncogene-encoded tyrosine kinases. Proc Natl Acad Sci USA 87: 3328–3332 2110361 10.1073/pnas.87.9.3328 PMC53893

[bib19] Kornberg L, Earp HS, Parsons JT, Schaller M, Juliano RL (1992) Cell adhesion or integrin clustering increases phosphorylation of a focal adhesion-associated tyrosine kinase. J Biol Chem 267: 23439–23442 1429685

[bib20] Kuwano H, Sumiyoshi K, Sonoda K, Kitamura K, Tsutsui S, Toh Y, Kitamura M, Sugimachi K (1997) Expression of p53 protein in glandular differentiation admixed with squamous cell carcinoma of the esophagus. Hepato-gastroenterology 44: 170–174 9058139

[bib21] Liotta LA, Steeg PS, Stetler-Steveson WG (1991) Cancer metastasis and angiogenesis: an imbalance of positive and negative regulation. Cell 64: 327–336 1703045 10.1016/0092-8674(91)90642-c

[bib22] Lipfert L, Haimovich B, Schaller MD, Cobb BS, Parsons JT, Brugge JS (1992) Integrin-dependent phosphorylation and activation of the protein tyrosine kinase p125^FAK^ in platelets. J Cell Biol 119: 905–912 1385445 10.1083/jcb.119.4.905 PMC2289696

[bib23] McCormack SJ, Brazinski SE, Moore Jr JL, Werness BA, Goldstein DJ (1997) Activation of the focal adhesion kinase signal transduction pathway in cervical carcinoma cell lines and human genital epithelial cells immortalized with human papillomavirus type 18. Oncogene 15: 265–274 9233761 10.1038/sj.onc.1201186

[bib24] Nishihira T, Hashimoto Y, Katayama M, Mori S, Kuroki T (1993) Molecular and cellular features of esophageal cancer cells. J Cancer Res Clin Oncol 119: 441–449 8509434 10.1007/BF01215923 PMC12201035

[bib25] Owens LV, Xu L, Craven RJ, Dent GA, Weiner TM, Kornberg L, Liu ET, Cance WG (1995) Overexpression of the focal adhesion kinase (p125FAK) in invasive human tumors. Cancer Res 55: 2752–2755 7796399

[bib26] Richardson A, Parsons JT (1995) Signal transduction through integrins: a central role for focal adhesion kinase? Bioassays 17: 229–236 10.1002/bies.950170309 7748177

[bib27] Schaller MD, Borgman CA, Cobb BS, Vines RR, Reynolds AB, Parsons JT (1992) pp125^FAK^, a structurally distinctive protein-tyrosine kinase associated with focal adhesions. Proc Natl Acad Sci USA 89: 5192–5196 1594631 10.1073/pnas.89.11.5192 PMC49256

[bib28] Schaller MD, Hildebrand JD, Shannon JD, Fox JW, Vines RR, Parsons JT (1994) Autophosphorylation of the focal adhesion kinase, pp125FAK, directs SH2-dependent binding of pp60src. Mol Cell Biol 14: 1680–1688 7509446 10.1128/mcb.14.3.1680-1688.1994 PMC358526

[bib29] Schlaepfer DD, Hanks SK, Hunter T, van der Geer P (1994) Integrin-mediated signal transduction linked to Ras pathway by GRB2 binding to focal adhesion kinase. Nature 372: 786–791 7997267 10.1038/372786a0

[bib30] Slack JK, Adams RB, Rovin JD, Bissonette EA, Stoker CE, Parsons JT (2001) Alterations in the focal adhesion kinase/Src signal transduction pathway correlate with increased migratory capacity of prostate carcinoma cells. Oncogene 20: 1152–1163 11313859 10.1038/sj.onc.1204208

[bib31] Takahashi K, Kanazawa H, Chan H, Hosono T, Takahara M, Sato K (1990) A case of esophageal carcinoma metastatic to the mandible and characterization of two cell lines (T.T and T.Tn) established from the oral tumor. Jpn J Oral Maxillofac Surg 36: 307–316

[bib32] Torres CM, Wang HH, Turner JR, Richards W, Sugarbaker D, Shahsafaei A, Odze RD (1999) Pathologic prognostic factors in esophageal squamous cell carcinoma: a follow-up study of 74 patients with or without preoperative chemoradiation therapy. Mod Pathol 12: 961–968 10530561

[bib33] Tremblay L, Hauck W, Aprikian AG, Begin LR, Chapdelaine A, Chevalier S (1996) Focal adhesion kinase (pp125FAK) expression, activation and association with paxillin and p50CSK in human metastatic prostate carcinoma. Int J Cancer 68: 164–171 8900422 10.1002/(sici)1097-0215(19961009)68:2<169::aid-ijc4>3.0.co;2-w

[bib34] Wang LS, Chow KC, Chi KH, Liu CC, Li WY, Chiu JH, Huang MH (1999) Prognosis of esophageal squamous cell carcinoma: analysis of clinicopathological and biological factors. Am J Gastroenterol 94: 1933–1940 10406262 10.1111/j.1572-0241.1999.01233.x

[bib35] Weiner TM, Liu ET, Craven RJ, Cance WG (1993) Expression of focal adhesion kinase gene and invasive cancer. Lancet 342: 1024–1025 8105266 10.1016/0140-6736(93)92881-s

[bib36] Xu LH, Owens LV, Sturge GC, Yang X, Liu ET, Craven RJ, Cance WG (1996) Attenuation of the expression of the focal adhesion kinase induces apoptosis in tumor cells. Cell Growth Differ 7: 413–418 9052982

